# Plant Extracts Rich in Polyphenols as Potent Modulators in the Growth of Probiotic and Pathogenic Intestinal Microorganisms

**DOI:** 10.3389/fnut.2021.688843

**Published:** 2021-07-30

**Authors:** Milica Milutinović, Suzana Dimitrijević-Branković, Mirjana Rajilić-Stojanović

**Affiliations:** Department of Biochemical Engineering and Biotechnology, Faculty of Technology and Metallurgy, University of Belgrade, Belgrade, Serbia

**Keywords:** prebiotics, medicinal herbs, polyphenols, antimicrobial activity, *Gentiana asclepiadea* L., *Hypericum perforatum* L., *Satureja montana* L., *Achillea millefolium* L.

## Abstract

Medicinal plants and their extracts contain substantial quantities of polyphenols. As metabolically active plant metabolites, polyphenols are food components with a wide range of biological activities. Given their poor absorbability in the digestive tract their activity toward the human host is typically mediated through interaction with intestinal microbes. As a result, polyphenols comprise a novel group of prebiotics. In this study, we tested the effect of five polyphenol-rich extracts from four medicinal herbs on the growth of probiotic and pathogenic microbes. The studied medicinal herbs were *Gentiana asclepiadea* L. (willow gentian), *Hypericum perforatum* L. (St. John's wort), *Satureja montana* L. (winter savory), and *Achillea millefolium* L. (yarrow). All these plants are traditionally used for the treatment of digestive problems. Extracts were prepared using safe solvent combinations. We tested the impact of addition of plant extracts on the growth of three probiotic lactobacilli and probiotic yeast *Saccharomyces boulardii*. The effect of addition of plant extracts to liquid media (concentration range 0.25–10 mg/mL) on the growth of probiotics, was tested *in vitro*. The antimicrobial activity of the extracts was tested against several opportunistic bacteria and yeast. St. John's wort, winter savory, and willow gentian extracts showed a stimulative effect on probiotic yeast growth, while the highest growth-stimulating effect was achieved when microwave-assisted yarrow extract was used in the concentration of 0.5 mg/mL. Under these conditions growth of *S. boulardii* was increased 130-fold. In addition, the yarrow extract stimulated the growth of *Lactiplantibacillus plantarum* 299v. The growth of two *Lacticasibacillus rhamnosus* strains was not stimulated by the addition of any extracts. Our results show that plant polyphenol-rich extracts can influence the growth of microorganisms that are typical members of the intestinal microbiota. For the first time we demonstrate that probiotic yeast growth can be stimulated by extracts of medicinal herbs, which when accompanied by suppression of *Candida* yeasts suggests a potential benefit of the treatment in diseases that are associated with fungal dysbiosis.

## Introduction

An individualized approach toward nutrition and medication is becoming increasingly important. While genetic and epigenetic host-factors contribute significantly to the individualized response to any compound that is taken up from the environment, another important factor of variation is human microbiota. The human microbiota is a complex ecosystem of microbes that inhabit various niches of the human body, with highest densities being on the skin, the oral cavity, and gastrointestinal tract. Of particular importance is colonic microbiota. It has been suggested that this anatomic part of the human body could be considered as a bioreactor in which an extremely dense microbial consortium is performing various functions that are important for systemic health (1). Gut microbiota is comprised of members of all three domains of life—Bacteria, Archaea and Eukarya; however, large individual differences hamper the complete description of the ecosystem (2). Despite its complexity and variability, recent research has clearly shown that interaction between gut microbiota and undigested food components, as well as medicaments, can yield various metabolites that could promote or jeopardize human health (3). Different types of studies contribute to resolving the puzzle between gut microbiota/food/health interactions, including *in vitro* tests, animal studies, and clinical trials. This is particularly important since many diseases, including obesity, diabetes, inflammatory bowel diseases, and colorectal cancer, have been associated with an imbalance in gut microbial composition, i.e., dysbiosis. Dysbiosis can occur due to decrease in the number of beneficial bacteria, overgrowth in detrimental species, and loss in microbial diversity (4, 5). One of the strategies promoting gut health is diet modification. Given that it has been shown that polyphenols and their metabolites have a positive impact on gut health (6), in this study, we aim to assess the impact of selected polyphenol-rich medicinal plant extracts on the growth of pathogenic and probiotic microorganisms, which in turn contributes to the modification of gut microbiota.

Polyphenols are plant metabolites, present in vegetables, fruits, spices, and medicinal plants with a wide range of biological activities, such as antioxidant, antimicrobial, anticancer, and anti-inflammatory (7, 8). Their dietary intake has been related to the prevention of some chronic and degenerative diseases, such as some types of cancers, inflammatory, cardiovascular diseases, diabetes, or neurodegenerative disorders like Alzheimer's and Parkinson's diseases. However, the exact mechanisms of polyphenol beneficial activity still remain to be defined (7–10). It has been estimated that <5–10% of the ingested polyphenols are resorbed in the small intestine, while the remaining compounds reach the large intestine where they are metabolized by the gut microbiota (8). Given their poor absorption in the digestive tract, their activity toward the human host is typically mediated through interaction with intestinal microbes, such that polyphenols comprise a novel group of prebiotics (11). *In vitro* studies suggest that polyphenols may exert a doubly positive effect, simultaneous inhibition of pathogens, and stimulation of beneficial bacteria (12). Beneficial species belonging to genera *Lactobacillus* and *Bifidobacterium* are often reported to thrive in the presence of polyphenols, whereas the growth of detrimental species is inhibited (13). Species of *Bifidobacterium* and *Lactobacillus* are usually used as probiotics, but some *Escherichia coli* and *Bacillus* strains, and the yeast *Saccharomyces boulardii* are used as well (14). *S. boulardii* is an extremely potent probiotic that is effective in preventing antibiotic-induced diarrhea, as well as in protecting the intestine from *Clostridium difficile* and cholera toxins (15). Additionally, the World Gastroenterology Organization recommends *S. boulardii* for the treatment of nine different intestinal diseases (14). The impact of polyphenol-rich medicinal plant extracts on the growth of probiotic or pathogenic microbes has not been assessed yet, while no previous study has assessed the effect of polyphenols on *S. boulardii* growth.

Many medicinal herbs have been used in folk medicine for treatment of gastrointestinal disorders (16). In this study, we evaluated the effect of different extracts of four medicinal plants that are traditionally used to treat digestive problems. *Achillea millefolium* L., commonly known as yarrow, has been used in traditional medicine for inflammatory and spasmodic gastrointestinal disorders, as an appetite-enhancing drug, for wound healing and diabetes (17, 18). *Hypericum perforatum* L., commonly known as St. John's Wort, has been used for poor appetite, difficulty sleeping, nervousness, the treatment of burns, skin ulcers, and cuts (19). *Satureja montana* L., commonly known as winter savory, is used for digestive complaints, such as diarrhea, and colic (20). Lastly, *Gentiana asclepiadea* L., commonly known as willow gentian, is traditionally used for improving appetite, digestive problems, and hepatitis infections (21). All listed medicinal plants have proven antimicrobial, antioxidant, anticancer, and anti-inflammatory properties (18, 19, 22–25). The major active compounds in the St. John's wort are flavonoids (quercetin, biapigenin, hyperoside, rutin, quercitrin, and isoquercitrin), phenolic acids (chlorogenic, caffeic, *p*-coumaric, and ferulic acid), naphthodianthrones (hypericin and pseudohypericin), and phloroglucinols (hyperforin and adhyperforin) (19, 22, 26). In the yarrow, predominant bioactive compounds are phenolic acids (chlorogenic, and 1,5-, 3,4-, 3,5- and 4,5-dicaffeoylquinic acids) and flavonoids (apigenin, luteolin, luteolin-7-O-glucoside, hyperoside, and rutin), tannins and sesquiterpene lactones (achillin and achillicin) (17, 18, 27). Winter savory has a diverse composition of secondary metabolites, such as phenolic monoterpene (carvacrol and thymol), phenolic acids (protocatechuic, syringic, vanillic acids caffeic, sagerinic, salvianolic, *p*-coumaric, ferulic, and rosmarinic acid), and flavonoids (luteolin, quercetin, rutin, epicatechin, catechin, and epicatechin) (24, 28–30). In the willow gentian, the major active compounds are secoiridoids (gentiopicrine, sweroside, and swertiamarine), flavonoids (isovitexin and homoorientin), phenolic acids, xanthone-C-glycosides (mangiferin), and alkaloids (25). Currently, forty-four medicinal plants are recommended in the European Union for treatment of gastrointestinal disorders, including the yarrow that can be used in mild, spasmodic gastro-intestinal complaints, and bloating (16). In this study, we assessed the effect of five extracts obtained from four medicinal plants on the growth of four selected probiotics, two strains of *Lacticaseibacillus rhamnosus* [previously *Lactobacillus rhamnosus* (31)], one *Lactiplantibacillus plantarum* [previously *Lactobacillus plantarum* (31)], and yeasts *S. boulardii*. The effect of plant extracts on the growth of opportunistic pathogens, *Enterococcus faecalis, Staphylococcus aureus, Escherichia coli, Pseudomonas aeruginosa, Listeria monocytogenes*, and *Candida albicans* was also tested.

## Materials and Methods

### Plant Material and Chemicals

The St. John's wort, winter savory, and yarrow were obtained from the Institute of Medicinal Plant Research “Dr Josif Pancic,” Pančevo, Serbia, while the willow gentian was obtained from “Bilje Borča,” Belgrade, Serbia.

A growth medium Tryptic Soy Broth (TSB), MRS broth, agar, and yeast extract were obtained from the Institute of Immunology and Virology, Torlak, Belgrade, Serbia. Resazurin was purchased from Acros Organics, Geel, Belgium, and ethanol was obtained from Zorka Pharma-Hemija d.o.o., Šabac, Serbia.

### Bacterial Strains and Culture Conditions

The following strains of bacteria and yeast were used: *Enterococcus faecalis* ATCC 29812, *Staphylococcus aureus* ATCC 25923, *Escherichia coli* ATCC 25922, *Pseudomonas aeruginosa* ATCC 27833, *Listeria monocytogenes* IM2002 (Institute of Meat Hygiene and Technology, Belgrade), *Lacticasibacillus rhamnosus* ATCC 7469, *Lacticasibacillus rhamnosus* GG (ATCC 53103), *Lactiplantibacillus plantarum* 299v, *Candida albicans* ATCC 10259, and *Saccharomyces boulardii* (*Saccharomyces cerevisiae* var. *boulardii* HANSEN CBS 5926), from the collection of the Department of Biochemical Engineering and Biotechnology of the Faculty of Technology and Metallurgy, Belgrade. Lactobacilli were grown overnight at 37°C in MRS broth in CO_2_ enriched conditions, while all other microorganisms were grown in TSB, supplemented with 0.6% w/v yeast extract at 37°C.

### Extraction of Polyphenols From Medicinal Plants

A total of five extracts were prepared from four medicinal plants, including yarrow, winter savory, St. John's wort, and willow gentian. The extracts were prepared either using standard procedures of the European Medicines Agency (EMA) or under optimized conditions for microwave-assisted extraction (MAE) that yields extracts with the highest polyphenol content. The optimization of the MAE extraction parameters was previously published for yarrow (32), while for the other used medicinal plants the manuscripts describing the optimization process are submitted/prepared for publication elsewhere.

Two extracts were prepared from St. John's wort. The first one was prepared using the optimized MAE procedure, in which three grams of St. John's wort was extracted for 40 s, with 150 mL of 30% ethanol at 170 W of microwave power. The second extract was prepared following the EMA procedure according to which ten grams of St. John's wort's waste powder was extracted with 100 mL of 50% ethanol in the dark at room temperature for 72 h (33). The yarrow extract was prepared using the optimized MAE under the following conditions 33 s of extraction time, 70% of ethanol concentration, 40 mL/g of liquid/solid ratio, and 170 W of microwave power (32).

The winter savory extract was prepared using the optimized MAE procedure for obtaining the extract with the highest polyphenol content in which three grams of plant powder were mixed with 60 mL of 55% ethanol and extracted for 80 s at 170 W of microwave power.

The willow gentian extract was prepared following the optimized procedure for obtaining an extract with the highest polyphenol content in which two grams of plant powder were extracted with 60 mL of 25% ethanol, in the dark, for 72 h.

After extractions, solids were separated from liquids using vacuum filtration and the ethanol was evaporated from all the extracts on a rotary evaporator (Büchi, Switzerland) at 150 mm Hg pressure and 60°C. The percentage of dry matter in the extract was measured on a moisture analyzer (Kern MLS-A, Balingen, Germany), and the extracts were stored at 4°C.

### Determination of Total Polyphenol Content

Total polyphenol content in the extracts was determined following the previously described method (32). The results were expressed as mg gallic acid equivalents (GAE)/g extract dry matter, using the gallic acid calibration curve. All experiments were performed in triplicate.

### Antimicrobial Activity

Antimicrobial activity of the extracts was determined using a broth microdilution method and resazurin as an indicator of microbial growth (34). For all pathogenic microorganism [three Gram-positive bacteria (*E. faecalis, L. monocytogenes*, and *S. aureus*), two Gram-negative bacteria (*E. coli*, and *P. aeruginosa*), and yeast *C. albicans*], and for all probiotic microorganisms (two strains of *L. rhamnosus, L plantarum*, and *S. boulardii*) the minimal inhibitory concentration (MIC) was determined.

In brief, 100 μL of TSB or MRS broth for lactobacilli was pipetted in all the wells of 96 well-microtiter plate. Afterward, 100 μL of plant extract stock solution (40 mg/mL) was pipetted in the first row and 2-fold serial dilution throughout the column was achieved by transferring 100 μL of the extract and nutrient broth mixture to the next row, so that the final volume in each well was 100 μL. The concentration of the plant extracts varied in the range from 0.016 to 20 mg/mL. Subsequently, 10 μL of microbial suspension was added to each well to achieve a final concentration of 5 × 10^6^ CFU/mL. Lastly, 10 μL of the resazurin solution (32.7 mM) was pipetted into all wells. Every plate contained a negative and a positive control. The negative control contained 10 μL of nutrient broth instead of the microbial suspension, while the positive control was prepared by mixing all solutions except the plant extract. The plates were incubated at 37°C for 24 h, and MIC values were determined visually based on color change. In the presence of growing cells purple resazurin is reduced to pink resorufin. The lowest concentration at which no color change occurred was considered as MIC. All tests were run in biological triplicate.

### Prebiotic Activity

The prebiotic activity of five plant extracts was tested in terms of growth stimulation potential. Liquid cultures using the plate count method were applied (35) and two *L. rhamnosus* strains, *L plantarum* 299v, and *S. boulardii* were tested. In short, fresh TSB or MRS (2 mL) were inoculated with an overnight growth culture of *S. boulardii* or lactobacilli (0.1 mL) to achieve a final concentration of 5 × 10^6^ CFU/mL. Plant extracts were added to the culture media to obtain a final concentration of dry matter in the range from 0.25 to 10 mg extract dry matter/mL, which are concentrations of extracts expected to be found under physiological conditions (33, 36). One control sample remained without any plant extract. After incubation (37°C for 24h), the samples were diluted with sterile normal saline and plated on TSA or MRS agar medium and incubated for 24 h at 37°C. The effect of plant extracts on lactobacilli species and *S. boulardii* was determined by comparing the number of colony-forming units (CFU) in the samples containing plant extracts and appropriate controls. All tests were run in biological triplicate.

### Statistical Analysis

Statistical analysis of the total polyphenol content in the extracts was performed in the program Origin Pro 8. All data were reported as mean ± standard deviation of three independent measurements. Significant differences between data were analyzed using analysis of variance (one-way ANOVA) and the Tukey test, with the criterion of *p* < 0.05.

Significant differences between the number of CFU on the plates with the plant extracts and the control were analyzed using a paired *t*-test with cut off value of *p* < 0.05 for significant difference. Previously, the Kolmogorov-Smirnov test was used to determine whether the data follows normal distribution, for which a paired *t-*test is a suitable tool for analysis.

## Results

### Total Polyphenol Content

Total polyphenol content in the tested extracts is presented in Table 1. Among all tested plants, the highest polyphenol content was identified in winter savory and the lowest content was determined in willow gentian. Comparing two St. John's wort extracts obtained using the MAE and EMA procedures, the total polyphenol content was higher in the MAE extract by 11.58%.

**Table 1:** Total polyphenol content in tested medicinal plant extracts.

	**Extract**				
	**Yarrow**	**St. John's wort** ^**a**^	**St. John's wort** ^**b**^	**Winter savory**	**Willow gentian**
TPC, mg GAE/g	204.61 ± 5.49^*^	282.01 ± 6.20^*^	252.75 ± 3.92^*^	386.75 ± 2.58^*^	19.21 ± 0.13^*^

*TPC, Total polyphenol content; GAE, gallic acid equivalent*.

a *St. John's wort extract obtained using the MAE procedure*.

b *St. John's wort extract obtained using the EMA procedure*.

* *Mean comparison was used to evaluate the significant difference among total polyphenol content in different plant extracts with the criterion of p < 0.05*.

### Antimicrobial Activity

Antimicrobial activity of all extracts was determined for three Gram-positive opportunistic pathogens (*E. faecalis, S. aureus*, and *L. monocytogenes*), two Gram-negative strains (*P. aeruginosa*, and *E. coli*) and yeast *C. albicans*. Antimicrobial activity was determined for probiotics as well, two *L. rhamnosus* strains, *L plantarum*, and *S. boulardii* (Table 2). Generally, Gram-positive bacteria were more sensitive to the plant extracts compared to Gram-negative. The most sensitive pathogen to the tested plant extracts was *L. monocytogenes*, as indicated by the lowest MIC value of 0.16 mg/mL. Only *S. aureus* and *L. monocytogenes* were inhibited by all the extracts. *E. coli* and *C. albicans* were sensitive only to winter savory and willow gentian, with MIC values of 5 and 20 mg/mL for *E. coli*, and 20 and 5 mg/mL for *C. albicans*, respectively.

**Table 2:** Antimicrobial activity of plant extracts.

	**Yarrow**	**St. John's wort** ^**a**^	**St. John's wort** ^**b**^	**Winter savory**	**Willow gentian**
**Microorganism**	**MIC, mg/mL**				
*S. aureus*	5	10	10	5	10
*L. monocytogenes*	1.25	2.5	2.5	0.16	20
*E. faecalis*	5	5	5	1.25	-
*E. coli*	-	-	-	5	20
*P. aeruginosa*	2.5	5	5	0.63	-
*C. albicans*	-	-	-	20	5
*L. rhamnosus* ATCC 7469	-	-	20	10	-
*L. rhamnosus* GG	-	-	10	10	-
*L. plantarum*	-	-	-	-	-
*S. boulardii*	-	-	-	20	20

a *St. John's wort extract obtained using the MAE procedure*.

b *St. John's wort extract obtained using the EMA procedure*.

*All tests were run in biological triplicate*.

Interestingly, the plant extracts showed higher inhibition to pathogens than to probiotics. Two St. John's wort extracts showed the same inhibition against pathogenic microorganisms, while the inhibition of probiotics growth was different. The MAE extract did not inhibit the growth of the tested probiotics, while the EMA extract inhibited the growth of two *L. rhamnosus* strains, with different MIC values (Table 2). This result suggested different chemical compositions of the two St. John's wort extracts.

Willow gentian suppressed the growth of *S. aureus, L. monocytogenes, E. coli*, and *C. albicans*, with MIC values ranging from 5 to 20 mg/mL, while it did not affect the growth of Gram-positive probiotics. It suppressed the growth of both yeasts, however, the MIC value for the *C. albicans* was 4-fold lower than the MIC value for *S. boulardii*.

The winter savory inhibited the growth of *L. rhamnosus* ATCC 7469 and *L. rhamnosus* GG, with equal MIC values. Antimicrobial activity toward the tested pathogens was stronger compared to the probiotic bacteria. The MIC values for pathogens varied in the range from 0.16 to 5 mg/mL, while it was 10 mg/mL for the two probiotics.

### Prebiotic Activity

**Figure 1:**
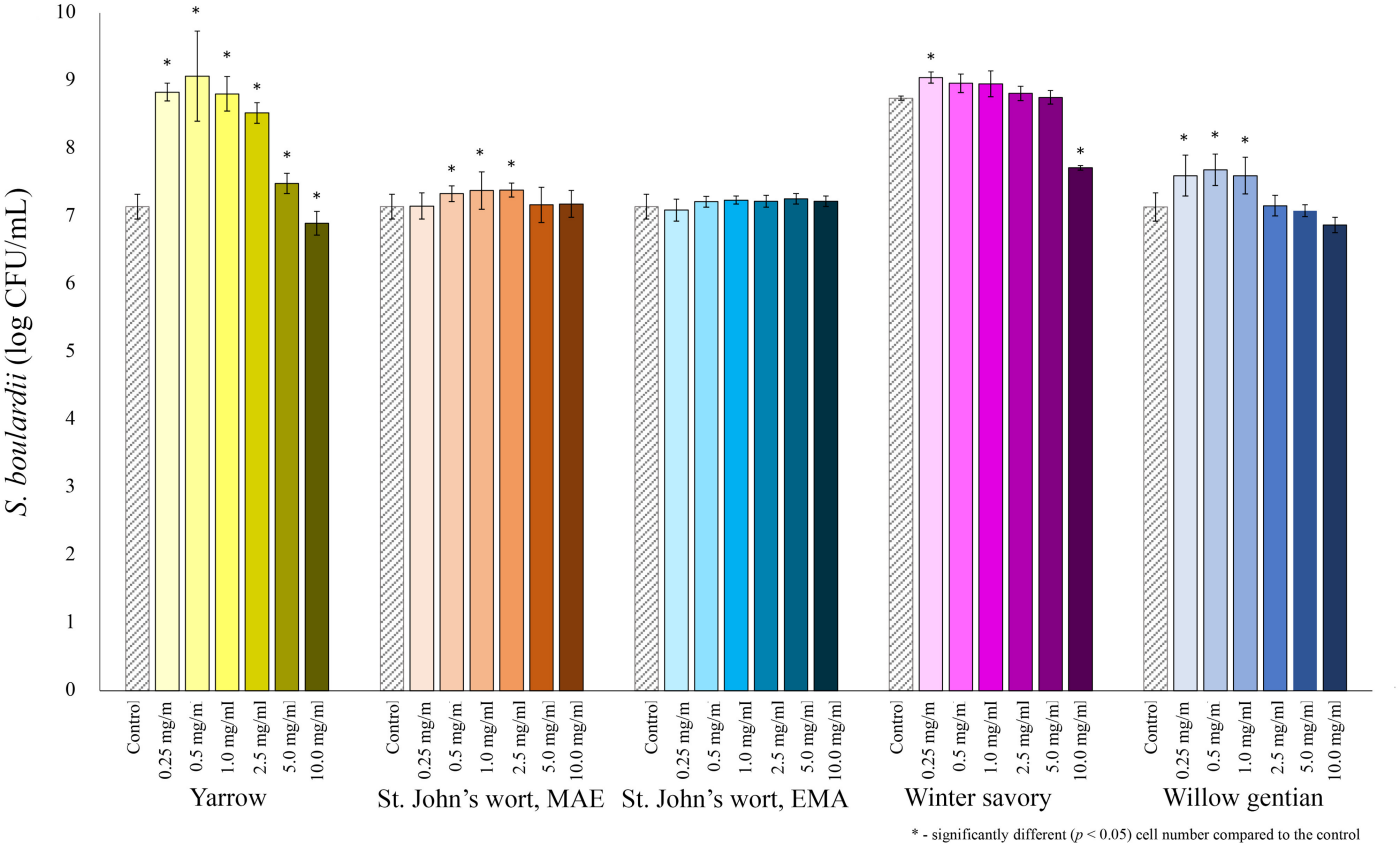
Growth of *S. boulardii* (log CFU/mL) after 24 h of growth at 37°C in the growth medium supplemented with plant extracts. Tests were run in biological triplicate.

The prebiotic potential of five plant extracts obtained from four medicinal plants was tested in terms of their ability to stimulate the growth of selected probiotics. The concentration of extracts in the growth medium was varied, and optimal concentration for growth stimulation of each extract–probiotic pair was identified. Among all tested probiotics, the yeast *S. boulardii* was the most stimulated by the addition of the yarrow MAE extract (Figure 1). The highest growth-stimulating effect was achieved when the MAE yarrow extract was added in the final concentration of 0.5 mg extract dry matter/mL of the growth medium. Under these conditions, the growth of *S. boulardii* was increased 130-fold. A significant 2-fold stimulation was also observed when the willow gentian or winter savory extracts were added to the growth medium in the final concentration of 0.5 or 0.25 mg/mL. However, both extracts inhibited the growth of *S. boulardii* when added in a concentration higher than 20 mg/mL. At this point, it should be noted that the growth performance of the control differed considerably when testing the winter savory and all other extracts. The tests were not performed in the same period, and for the winter savory a new lot of the same *S. boulardii*, strain was used. The strain from the new lot grew better in the control nutrient broth.

**Figure 2:**
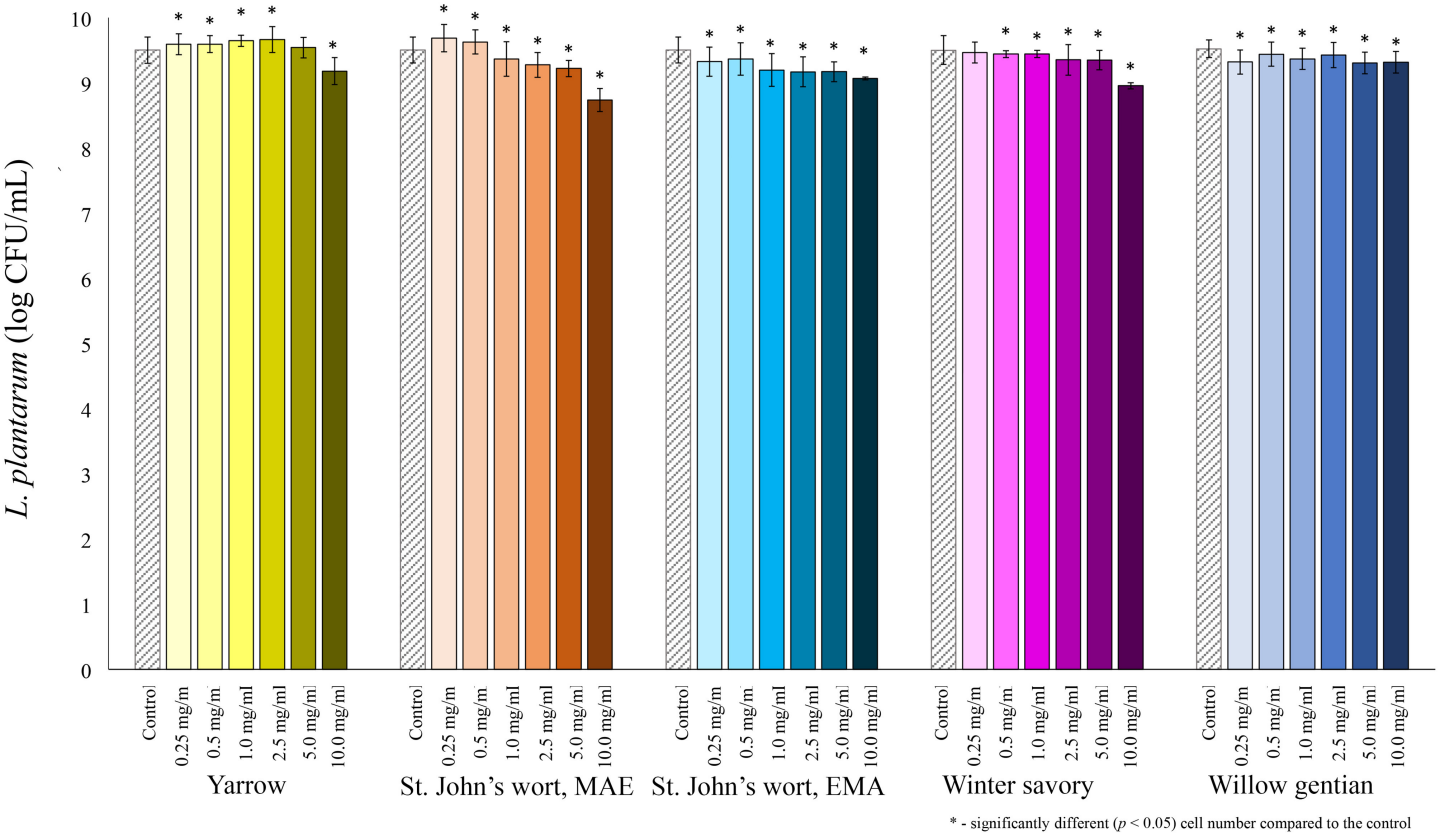
Growth of *L. plantarum* (log CFU/mL) after 24 h of growth at 37°C in the growth medium supplemented with plant extracts. Tests were run in biological triplicate.

**Figure 3:**
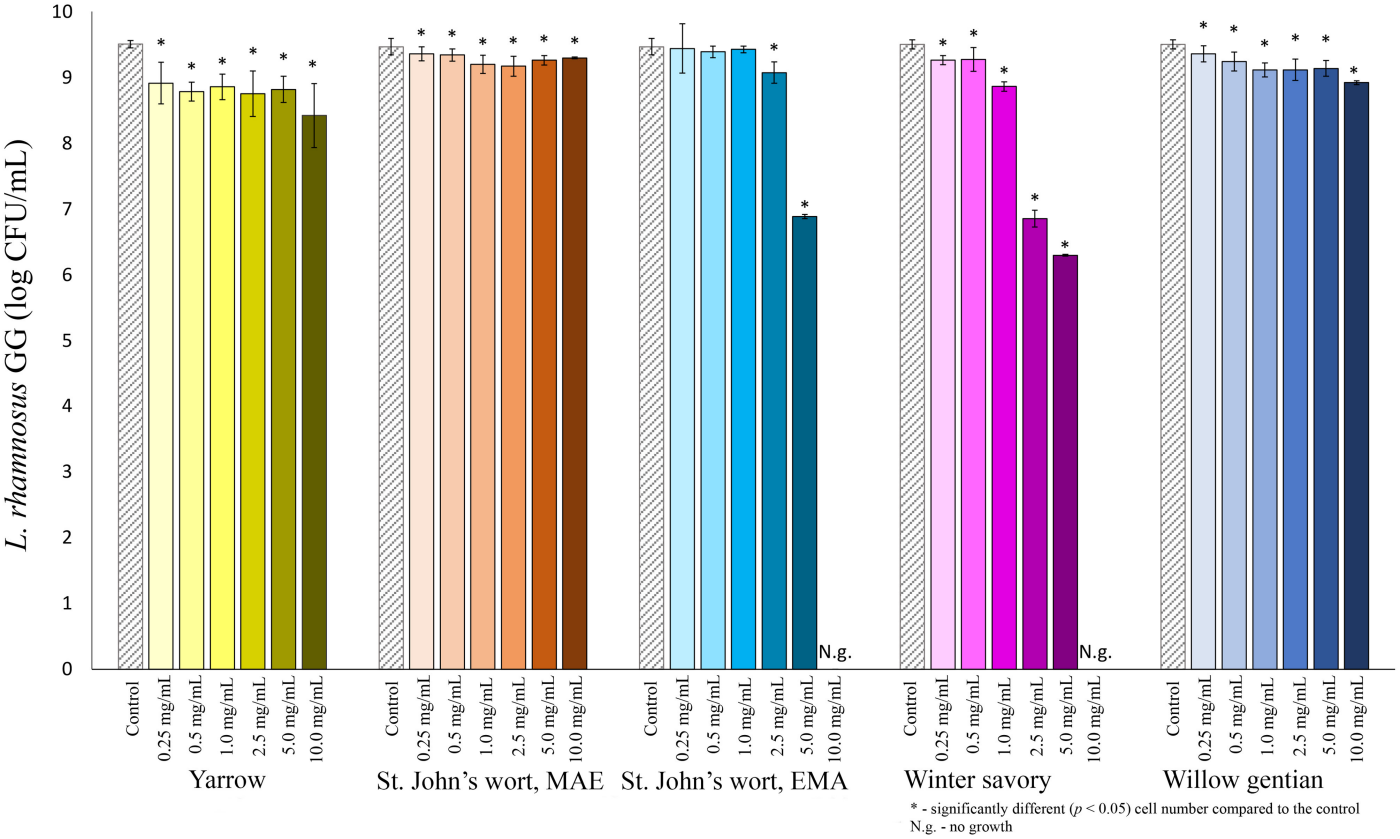
Growth of *L. rhamnosus* GG (log CFU/mL) after 24 h of growth at 37°C in the growth medium supplemented with plant extracts. Tests were run in biological triplicate.

**Figure 4:**
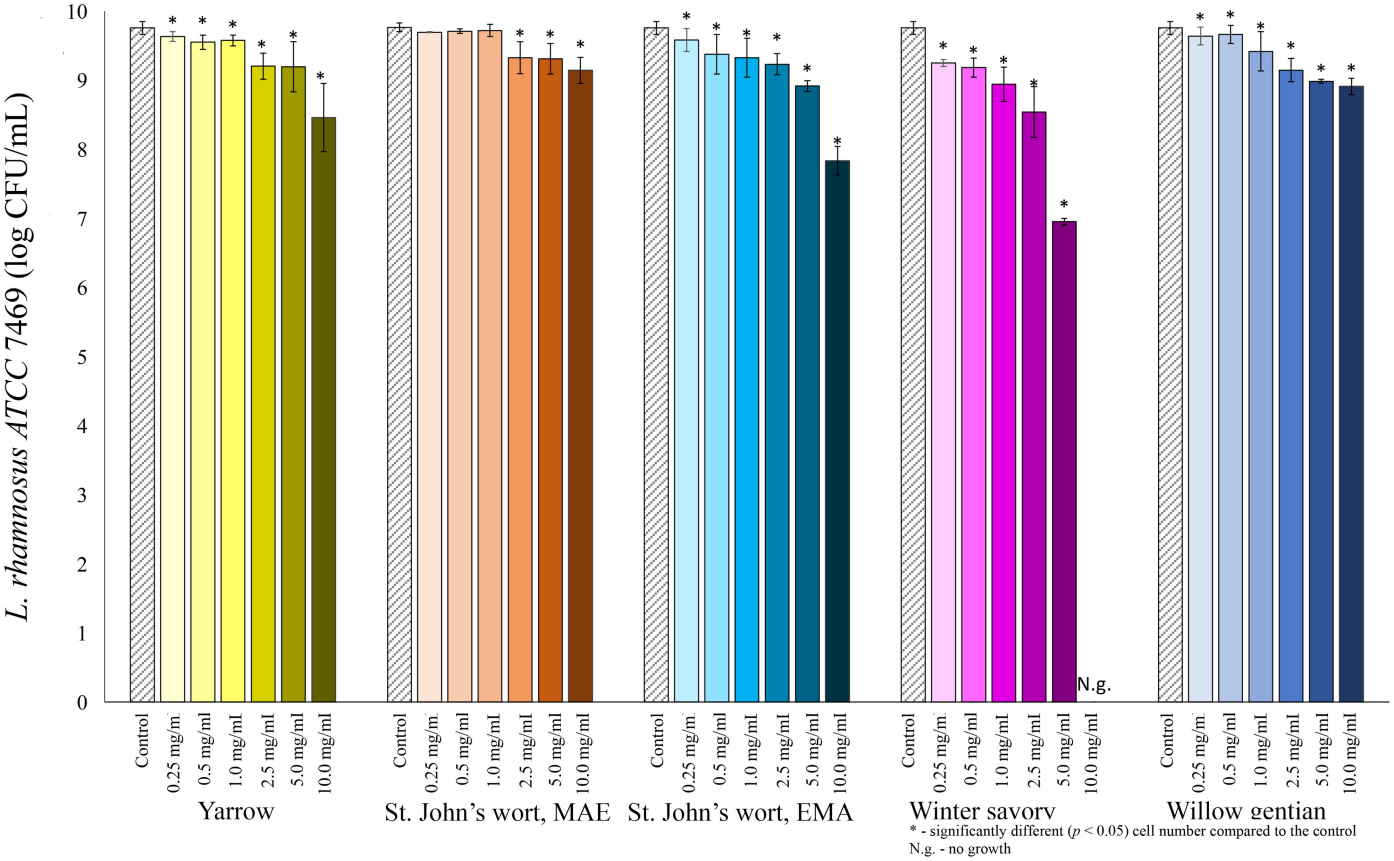
Growth of *L. rhamnosus* ATCC 7469 (log CFU/mL) after 24 h of growth at 37°C in the growth medium supplemented with plant extracts. Tests were run in biological triplicate.

Among lactobacilli, the growth of *L. plantarum* was stimulated with the addition of the yarrow extract, and the St. John's wort MAE extract (Figure 2). Growth stimulation was achieved when the concentration of yarrow MAE and St. John's wort MAE extracts was in the range 0.25–2.5 and 0.25–0.5 mg/mL, respectively. When the concentrations of the extracts further increased, the cell count significantly decreased compared to the control. The growth of two *L. rhamnosus* strains was not stimulated by the addition of any of the five plant extracts in the tested concentrations (Figures 3, 4).

## Discussion

The present study provides new findings on the impact of yarrow, St. John's wort, winter savory, and willow gentian extracts on the growth of selected pathogens and probiotics. We demonstrate that these medicinal plants, which are traditionally used for the treatment of digestive problems, could suppress the growth of pathogens, while the presence of the same concentration could stimulate the growth of probiotics. Hence, one potential mechanism of the beneficial effect of medicinal plants could be modulation of the gut microbiota composition and/or activity.

It is important to emphasize that in this study the growth stimulation of probiotics and antimicrobial activity against pathogens was achieved with a concentration of medicinal plant extracts that can be expected to be found under physiological conditions. We could evaluate the intake only for St. John's extract prepared according to EMA procedure, as only for this extract, official recommendations exist from among all tested extracts (33). Daily recommended intake of St. John's extract is 10 ml. Based on extract concentration, this would require intake of ~180 mg of extract dry matter. Given that the exact dilution factor under physiological conditions can be only estimated, it is important to perform *in vivo* human intervention studies. Nevertheless, we would like to note that an estimated dilution of the maximal dose of St. John's EMA extract in 200 mL would facilitate the maximal concentration tested in our growth stimulation experiments. Furthermore, one intervention on healthy subjects, showed that intake of cocoa-derived flavanols in a dosage similar to that achieved following EMA recommended daily intake of St. John's extract, was sufficient to induce changes in gut microbiota (36).

The selected medicinal plants have proven antioxidant, antimicrobial, and anti-inflammatory properties, among others, due to the presence of bioactive compounds, such as polyphenols. It is known that the concentration of polyphenols in plants depends on the species of plant, environmental conditions, rainfall and ultraviolet radiation, geographical origin, and storage conditions (37). Further, the total polyphenol content in the extract depends on the extraction technique and solvent used in the process. Ethanol and water mixtures were used in the preparation of all five extracts. Ethanol has GRAS (generally regarded as safe) status and can be used for preparation of extracts that are safe for human consumption (38). Of the four tested plants, winter savory is the richest in polyphenolic compounds, followed by St. John's wort, yarrow, and willow gentian. These findings are consistent with literature data (21, 29, 39, 40). Comparing the extracts obtained from the same plant material, but with different extraction techniques, it can be concluded that MAE was more efficient than the maceration. Using modern extraction techniques, such as MAE, the yield of polyphenols can be increased, while extraction time and solvent usage are reduced (41). Additionally, polyphenol composition may differ when two extraction techniques are used (41). This was most likely the case for the two St. John's wort extracts, as indicated by their different biological activity.

Of the four tested plants, winter savory contained the highest concentration of polyphenols and this extract had the highest antimicrobial effect on all six tested pathogens. The highest antimicrobial activity was against *L. monocytogenes*, while the lowest was against *C. albicans* (Table 2). The two St. John's wort extracts inhibited the growth of four pathogens with equal MIC values, while the effect on probiotics was different. While the EMA extract inhibited the growth of two *L. rhamnosus* strains, the MAE extract stimulated the growth of *S. boulardii* and *L. plantarum*, indicating that there is a difference in the composition of polyphenols in the two extracts. All five extracts showed stronger antibacterial activity against Gram-positive bacteria than against Gram-negative ones (Table 2). Generally, the antibacterial effect of plant extracts was usually more effective against Gram-positive than Gram-negative bacteria (13). The exact mechanism of action of polyphenols as antimicrobial agents is not yet fully understood. Polyphenols could react with the cell membrane, inactivate essential cellular enzymes, while lipophilic flavonoids could disrupt microbial membranes and cause the loss of cell's macromolecules and subsequent cell death (42–44). Also, a synergistic effect of polyphenolic compounds present in the plant extract contributes to higher antimicrobial activity compared to a single compound's reaction, which may be due to the different mechanisms of action of each of the active components (45). Among all tested extracts, the willow gentian showed the highest antimicrobial activity toward *C. albicans* (Table 2), while the concentration of polyphenols in this plant extract is lower than in all other plant extracts. Besides polyphenols, the major compounds in willow gentian are secoiridoids (gentiopicrin), and it has been reported that the gentiopicrin inhibited the growth of Gram-negative, Gram-positive pathogen bacteria, as well as yeast *C. albicans* (46). Additionally, the predominant constituents of St. John's wort besides polyphenols are naphthodianthrones (hypericin and pseudohypericin) and phloroglucinols (hyperforin and adhyperforin) (26). It has been reported that hypericin and hyperforin are components responsible for the antimicrobial activity of the St. John's wort extract (26). Antimicrobial activities of all four medicinal plants have been previously published (47–54). However, in most of the studies, different and often toxic solvents were used for the preparation of extracts. As stated above all five extracts analyzed in this study were prepared using GRAS solvents (ethanol and water mixtures), which makes the extracts safe for human consumption (38). Compared to our results, only Mekinić and colleagues (47) reported higher antimicrobial activity of ethanol yarrow extract, with the MIC values for *L. monocytogenes* and *S. aureus*. Moreira et al. (29) reported lower antimicrobial activity of winter savory extract compared to our results. Their extract inhibited the growth of *S. aureus* and *L. monocytogenes* with MIC values of 20 mg/mL, which are 4 and 125 times higher than the MIC values obtained in this study, respectively. Since the total polyphenol content obtained in this study is only slightly higher than in Moreira et al. (24.62 ± 0.16 vs. 23.43 ± 4.33 mg GAE/g plant dry weight), it can be expected that the MAE enhanced the extraction of more potent antimicrobial compounds. In addition, the MAE winter savory extract inhibited the growth of *E. coli*, which is in contrast to previous reports (20, 29). Moreover, the antimicrobial activity of medicinal plants depends on the date of plant collection. Borchardt et al. showed that a St. John's wort plant sample collected in August inhibited the growth of some pathogens, while the sample collected in July had no antimicrobial activity (55). This indicates that polyphenol content of medicinal plant extracts and consequent biological activity is affected by numerous factors.

The prebiotic effect of polyphenols from medicinal plants has not been assessed previously, but the influence of some plant extracts rich in polyphenols on the growth of pathogen and probiotic bacteria has been studied (35, 56–58). In our study, among all tested extracts, the highest growth-stimulating effect showed yarrow MAE extract on the yeast *S. boulardii*. With the dosage of 0.5 mg/mL in the growth medium, the maximal 130-fold stimulation of *S. boulardii* was achieved. The addition of St. John's wort MAE, willow gentian, and winter savory extracts caused an increase of *S. boulardii* growth, but not as strong as yarrow MAE extract. The exact mechanism by which polyphenols increase the growth of probiotics is not known. Most of the published papers discuss the effect of the plant extract on the pathogens and probiotic species of *Lactobacillus* and *Bifidobacterium*. To the best of our knowledge, the influence of plant extracts, or polyphenol compounds, on the growth of *S. boulardii* was not previously studied. Both winter savory and willow gentian stimulated the growth of *S. boulardii* ~2-fold; however, the concentration of polyphenols between these two extracts was significantly different, indicating that other bioactive compounds from willow gentian could stimulate the growth of the probiotics. As reported before, the major compounds in willow gentian extracts are secoiridoids (gentiopicrin), besides polyphenols; however, there is no data in the literature on their influence on the growth of probiotic bacteria or yeast (21). It has been reported that some species of lactobacillus are able to metabolize gentiopicrin by the action of their β-glucosidase, producing aglycon and sugar moiety (59). Given the impact that the tested plant extracts showed on the pathogenic yeast *C. albicans*, it can be concluded that consumption of the selected plants could have a beneficial effect on the *Candida/Saccharomyces* ratio, which could be relevant for treatment of fungal dysbiosis that was reported for inflammatory bowel disease and the diarrhea predominant irritable bowel syndrome (60, 61). Therefore, our data indicates that tested medicinal plant extracts could be used in precise nutrition/medical treatment of conditions affected by fungal dysbiosis. However, it should be noted that particular impact on the gut microbiota can be determined only in clinical trials.

Of all tested probiotic bacteria, only *L. plantarum* was stimulated by the addition of two plant extracts. The highest stimulation, 1.5-fold was achieved by the addition of St. John's wort extract in the final concentration of 0.25 mg/mL, followed by the yarrow MAE extract. Additionally, the yarrow MAE extract induced much higher, 6-fold growth stimulation of one more strain of *L. rhamnosus, L. rhamnosus* A71, with a concentration of 2.5 mg/mL in the growth medium (62), while it suppresses the growth of three Gram-positive and one Gram-negative bacteria, with the MIC values from 1.25 to 5 mg/mL. This suggested a potential use of the yarrow MAE extract as a dietary supplement that could initiate the change of the gut microbiota ecosystem in the direction of dysbiosis repair, contributing to increase in the relative abundance of probiotics and decrease in the relative abundance of opportunistic pathogens. Similar results could be expected for other extracts, such as winter savory, which showed the lowest growth-stimulation on tested probiotics among all four plants. It also inhibited the growth of *L. rhamnosus* ATCC 7469 and *L. rhamnosus* GG with equal MIC values, which was higher than the MIC values for pathogenic microorganisms. While our data is promising, it should be emphasized that the exact impact on the gut microbiota ecosystem can be assessed properly only in clinical trials. Based on published data on the effect of polyphenols on the growth of probiotic bacteria, one possible explanation for the stimulation of growth is that probiotics could metabolize polyphenols during growth (63, 64). Some microorganisms can hydrolyze O-glycosylated polyphenols to aglycone and glucose, which they can use for their growth, as a sole source of energy and carbon (65–67). Also, *L. plantarum* can degrade *p*-coumaric, caffeic, ferulic, coumaric, gallic and protocatechuic acid to obtain energy (13, 68, 69). For example, Duda-Chodac reported that the aglycons naringenin and quercetin inhibited the growth of *Lactobacillus* sp., while rutin showed slight stimulation on the growth (70). Rutin, *p*-coumaric, and chlorogenic acids are found in the St. John's wort and yarrow extracts which could explain their stimulatory role on the *L. plantarum* growth. Even though the bioactivity of the plant is related to its major components, the combined effect of all components in the plant can be more important for the effect of the herbal products than the activity of specific compounds (24). Therefore, the prebiotic activity could be due to the combined effect of all compounds present in the extract (24). Moreover, polyphenols in extracts could reduce oxidative stress in the medium caused by metabolic activities, thus providing better conditions for the growth of probiotic microorganisms (35).

## Conclusions

The present work provides new findings on the influence of extracts from four medicinal plants—yarrow, winter savory, St. John's wort, and willow gentian—on the growth of probiotic and pathogenic microorganisms. For the first time, it was shown that polyphenol-rich medicinal plants could stimulate the growth of *S. boulardii* while suppressing the growth of pathogenic *Candida* yeast. This opens the possibility for the application of these plants in the treatment of fungal dysbiosis which could be particularly relevant for patients suffering from conditions in which the *Candida*/*Saccharomyces* disturbed ratio has been reported. Among all tested plants, the strongest antimicrobial activity was shown for the winter savory extract, which inhibited the growth of all tested pathogens. Interestingly, all tested plant extracts showed the ability to promote the growth of some of the tested probiotics and suppress the growth of some of the tested pathogens. Based on the provided data it can be speculated that medicinal plants, and particularly their polyphenol-rich extracts, could have the ability to modulate gut microbiota *in vivo*. Given that gut microbiota shows tremendous interindividual variation, and that polyphenol effects on microbial species might even be strain-dependent (65), it can be expected that the effect of medicinal herbs on microbiota will be highly individualized. This could potentially explain the high variability in response to the plant bioactive compounds, which is well-documented (71). Our results provide the first hint of the important interaction between medicinal herbs polyphenols and fungal and bacterial constituents of gut microbiota. Further studies, primarily ones based on clinical trials, will elucidate their role in microbiota-dependent personalized nutrition and medical treatment.

## Data Availability Statement

The original contributions presented in the study are included in the article/supplementary material, further inquiries can be directed to the corresponding authors.

## Author Contributions

MR-S created the main conceptual ideas. MM performed the experimental work and statistical analysis. MM, SD-B, and MR-S contributed to the organization and writing of the manuscript. All authors contributed to the article and approved the manuscript for publication.

## Conflict of Interest

The authors declare that the research was conducted in the absence of any commercial or financial relationships that could be construed as a potential conflict of interest.

## Publisher's Note

All claims expressed in this article are solely those of the authors and do not necessarily represent those of their affiliated organizations, or those of the publisher, the editors and the reviewers. Any product that may be evaluated in this article, or claim that may be made by its manufacturer, is not guaranteed or endorsed by the publisher.
